# Exogenous Hormones and Their Clinical Implications for the Development and Growth of Meningioma Tumours

**DOI:** 10.3390/jcm15145560

**Published:** 2026-07-15

**Authors:** Holly Roy, Marios Stavrakas, Samiul Muquit

**Affiliations:** 1 Department of Neurosurgery, University Hospitals Plymouth NHS Trust, Plymouth PL6 8DH, UK; s.muquit@nhs.net; 2 Department of ENT, University Hospitals Plymouth NHS Trust, Plymouth PL6 8DH, UK; m.stavrakas1@nhs.net

**Keywords:** meningioma, menopausal hormonal replacement therapy, oral contraceptives, cyproterone acetate

## Abstract

Meningiomas are the most common central nervous system tumour and are associated with significant morbidity. Mounting evidence implicates the role of exogenous hormones in meningioma development and growth. This has led to increasing pressure on healthcare professionals to understand the risk profile of different hormonal compounds with regards to meningioma incidence and growth. This is particularly relevant when managing patients with pre-existing meningioma tumours or with one or more risk factors for meningioma formation. The aim of this review was to summarise existing evidence from clinical studies (cohort and case–control) concerning the risk of meningioma associated with different types of exogenous hormones and the associated meningioma characteristics. The literature review identified over 30 cohort and case–control studies published between 2003 and 2026. Studies demonstrated a mixed risk profile of broadly defined hormonal replacement therapy and hormonal contraceptives; however, many of these studies did not capture specific information about duration, dose or medication type. Risk associated with synthetic progestin-containing compounds such as depot medroxyprogesterone and desogestrel contraceptives was higher and related to duration of use. Highly potent synthetic progestins including cyproterone acetate (CPA) carried the strongest risk profile and showed a strong association with multiple meningiomas and anterior/middle skull base location. In conclusion, there is strong evidence for a link between meningioma incidence and highly potent synthetic progestins such as CPA, but further evidence is needed regarding menopausal hormone therapy and broadly defined oral contraceptives, as well as the doses and durations that are associated with clinically relevant risk. There is a space for translational research in this area to better understand the molecular basis underlying the relationship between hormones and meningioma growth.

## 1. Introduction

Meningiomas are the most common type of benign brain tumour [1]. There are 15 subtypes, of which nine are classified as WHO grade 1, three as WHO grade 2 and one as WHO grade 3 [2,3]. While the majority of meningiomas are grade I, morbidity arises due to mass effect resulting in compromise to functional neural structures (including cranial nerves and eloquent cortex), raised intracranial pressure, and seizures. Morbidity can also be associated with the complications of surgery (particularly when tumours are adjacent to critical neurological and vascular structures) or radiotherapy. Established risk factors for meningioma development include genetic syndromes (including NF2-associated schwannomatosis) [4], radiation exposure [5], female sex [6] and obesity [7,8]. The role of sex hormones in the development and growth of meningiomas is not fully understood, but observations point towards a tumour-promoting effect. For example, meningiomas can grow during pregnancy and regress after delivery [9] and are associated with other conditions linked to levels of sex hormones such as breast cancer [10,11]. In addition, meningiomas often express sex hormone receptors, including progesterone receptors, androgen receptors and oestrogen receptors [12,13]. Progesterone receptor expression occurs in 60–90% of intracranial meningiomas and may be more common in skull base meningiomas [13,14].

Cumulative evidence has linked the potent progestin cyproterone acetate (CPA) to meningioma growth, particularly in the region of the anterior and middle skull base, which has led the UK Medicine and Healthcare Products Regulatory Agency (MHRA) to issue guidance that CPA is contraindicated in patients with a current or previous meningioma (with the exception of its use in palliative prostate cancer treatment). The guidance also states that treatment should be immediately and permanently stopped if a patient develops a meningioma while on CPA [15]. Similar guidance has been issued by the European Medicine Association (EMA) [16], and as the risk of meningioma with CPA use is cumulative and dose dependent, prescribers are encouraged to consider using the lowest effective dose for the shortest duration of time. This has led to questions about the potential risk of meningioma formation/growth with the use of other hormonal-based therapies including hormonal contraceptives and menopausal hormone replacement therapy, particularly regarding their cumulative effect, their interaction with other meningioma risk factors, and whether they should be stopped if a meningioma is diagnosed. The aim of this study is to evaluate areas of strong, emerging and weaker evidence concerning the use of exogenous hormones and the risk of meningioma and to identify directions for future research to better inform clinical guidance and decision making (Table 1).

## 2. Methods

A literature search was carried out in PubMed (MEDLINE), Scopus and EMBASE databases, using the following search terms: cyproterone acetate, nomegestrol acetate, chlormadinone acetate, medroxyprogesterone acetate, depot medroxyprogesterone acetate, desogestrel, levonorgestrel, oral contraceptives, hormone replacement therapy, menopausal hormone therapy, progestins and meningioma. Inclusion criteria were (1) observational studies (cohort and case–control) published between 1 January 2000 and 3 March 2026; (2) stated study objectives concerning meningioma and interaction with HRT/MHT, oral contraceptives or cyproterone acetate or another high-dose progestin; (3) English language; and (4) human only. Papers which did not meet the inclusion criteria were not studied further.

In papers which met the inclusion criteria, information was extracted relating to the following variables: study design; numbers of patients included; type of hormonal contraceptive/menopausal hormone therapy including dose and route of administration; association with meningioma; and additional information about meningioma subtype, grade, genetics, multiplicity and hormone receptor status if available. Full data extraction is available in the Supplementary Materials (Supplementary File S1). Quality assessment for all papers was carried out using the Newcastle-Ottawa Scale, and results of this assessment along with a full list of finally included studies are available in the Supplementary Materials (Supplementary File S2). Newcastle-Ottawa Scale assessment was performed manually for each individual paper with a sample of papers cross-checked by a second reviewer. Where there were disagreements, these were discussed and scores were modified accordingly.

## 3. Results

**Figure 1:**
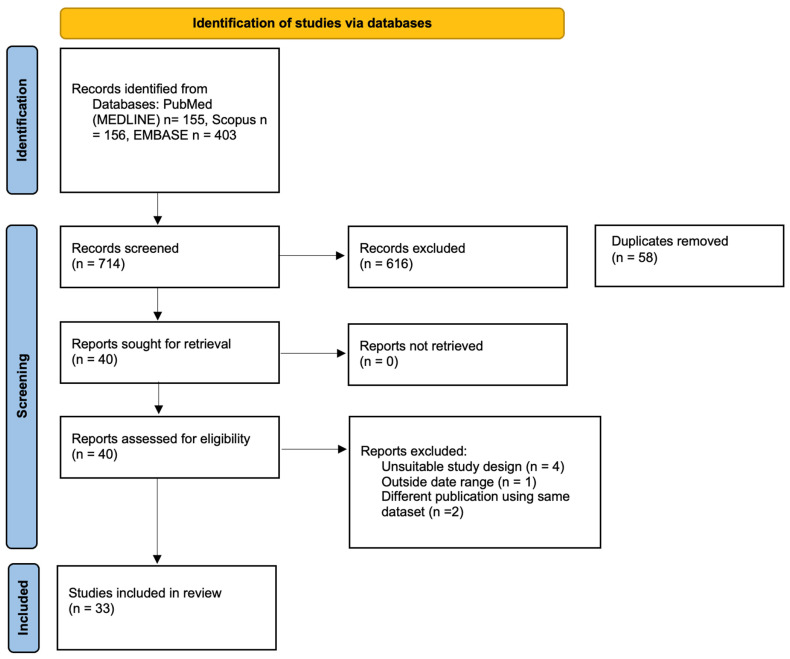
PRISMA style flow chart to demonstrate process of study selection.

A total of 714 papers were identified across the databases. Abstracts were screened for suitability and compliance with the study objectives and inclusion criteria, leading to the exclusion of 616 records and removal of 58 duplicates. Duplicate removal was carried out manually. The 40 remaining papers were assessed in detail, and 7 further papers were excluded, leading to a total of 33 papers being included within the study (see Figure 1 for PRISMA style flow diagram [17] and Supplementary File S2 for a list of the final included studies).

Nine studies contained data regarding highly potent progestins such as (CPA), nomegestrol acetate (NOMAC) and chlormadinone acetate (CMA) [18,19,20,21,22,23,24,25,26]; 8 studies contained data concerning other progestin-related formulations [27,28,29,30,31,32,33,34]; 12 studies contained information risk with hormonal replacement therapy [35,36,37,38,39,40,41,42,43,44,45,46]; and 13 papers contained data about meningioma risk with broadly defined oral contraceptive use [27,36,39,40,41,42,44,45,46,47,48,49,50].

Using the Newcastle-Ottawa Scale for bias assessment, the median score for cohort studies was 7/9 (range 5–9) and the median score for case–control studies was also 7/9 (range 5–9).

### 3.1. High-Potency Progestins

The strongest evidence identified in this review was that which explored the association between meningioma and the use of potent synthetic progestins CPA, NOMAC and CMA. These three compounds were considered to be in the high-risk group of compounds following a classification used in Devalckeener et al. (2022) [51].

Nine studies were identified which specifically explored the association between meningioma formation and the use of potent synthetic progestins CPA [20,21,22,23,24,25,26], NOMAC [18,25] and CMA [19,25]. These studies consistently found risk of meningioma to be increased with the use of potent progestins. Risks were highest for CPA, then NOMAC, then CMA; for example, Hoisnard et al. [25] reported odds ratios of meningioma with current use of CPA of 18.3 (95% CI 16.0–21.1), with current use of NOMAC of 4.7 (95% CI 4.3–5.1), and with current use of CMA of 3.3 (95% CI 3.0–3.6).

There also appeared to be a clear dose dependency in the relationship between progestin use and meningioma formation [18,20,21,24,25]. For example, according to Mikkelsen et al. in their nationwide prospective register-based cohort study [20], adjusted hazard ratios for CPA users exposed to 0.1–10 g CPA compared to non-users were 7.0 (95% CI 3.1–15.6), whereas for individuals exposed to >10 g the adjusted hazard ratios were 19.2 (95% CI 10.3–35.8). Some cases of CPA-associated meningiomas were shown to regress after CPA withdrawal [24].

Data also demonstrated a strong link between CPA and an anterior and middle skull base location of the tumour [21,23,25], with a similar pattern likely for NOMAC use [18,25] and CMA [25]. There was also a strong link between CPA and multiple meningiomas [22]. High PR expression was described in CPA-associated meningiomas in one study [22], and no single genetic mutation was consistently associated with CPA or progestin-related meningiomas.

### 3.2. Depot Medroxyprogesterone Acetate and Other Contraceptive Progestins

Our review identified emerging evidence that other contraceptive-related progestins may also contribute to meningioma risk. Our search identified eight studies published between 2000 and 2026 exploring the association between progestin-based formulations and meningioma.

The strongest evidence for this is in recent studies looking at dMPA. dMPA was demonstrated in all studies, which specifically examined its role, to increase the risk of meningioma [29,30,31,32] and to do so with greater probability with prolonged exposure [29,31]. For example, Xiao et al., 2025 [29] showed a higher probability of meningioma diagnosis in dMPA users compared to controls (RR 2.43 (95% CI 1.77–3.33)), with RR increasing to 3.90 (95% CI 1.95–7.81) with >6 years exposure. Nevertheless, the number needed to harm using dMPA was 1152 patients, and with oral MPA was 3020 patients [29], which the authors felt could be considered to represent a low clinical risk overall. Reynolds et al. also evaluated oral and dMPA separately and found that while oral MPA was not associated with increased meningioma risk, long-term exposure to dMPA injections (more than one year) resulted in an increased association with meningioma (OR 1.81, 95% CI 1.14–2.89) [31]. A Swedish register-based case–control study [30] showed that the odds ratio of meningioma for women prescribed hormonal contraceptives for equal to or more than 1 year before the index date was 1.76 (95% CI 1.53–2.03) and that for women prescribed contraceptives containing MPA the OR was 5.49 (95% CI 4.51–6.67). Frey et al. (2025) [32] described a significantly increased association between meningioma and MPA use compared to ethinylestradiol-levonorgestrel use; the RR for MPA use compared with EE-LNG was 3.55 (95% ICI: 1.85–6.55).

In terms of other progestin-based hormonal contraceptives, Roland et al. [33] found that intracranial meningioma was linked with the use of desogestrel 75 µg for more than five continuous years but that there was no association between meningioma and levonorgestrel intrauterine system use (alone or combined with oestrogen). According to their numbers needed to perform harm evaluation, 67,287 women would need to use desogestrel 75 ug to result in one attributable meningioma requiring surgery (compared to a number needed to harm of 518 women concerning CPA use). This study also described an association between desogestrel use and meningiomas occurring in multiple locations, as well as between desogestrel use and an anterior or middle skull base location [33].

### 3.3. Menopausal Hormone Therapy

Twelve studies were identified which looked at the association between menopausal hormone replacement therapy and meningioma. Nine were case–control studies and three were cohort studies.

Three studies reported no association between HRT and meningioma diagnosis [36,40,45]. Two of these studies included only surgically treated meningiomas [36,40]. In a relatively small case–control study of incidental meningioma, Dresser et al. (2020) [37] concluded that meningiomas were smaller at diagnosis and follow-up in patients taking oestrogen-based HRT compared with the no-HRT group, with a lower absolute growth rate and clinico-radiological progression free survival [37] implying a protective effect of oestrogen-based HRT.

Eight studies reported modest associations between HRT and meningioma, particularly when HRT was used for longer durations. Ever-use of HRT was associated with a higher risk of meningioma than non-use of HRT in a case–control study based in Denmark [38]. This study recruited 924 cases and 6122 controls. The strongest association was for longer duration of HRT use (>10 years) and in combined oestrogen-progesterone formulations [38]. Jhawar et al. 2003 [41] found a higher risk of incident meningioma in pre-menopausal women taking HRT (RR 2.48, 95% CI 1.29–4.77) and post-menopausal current users of HRT (RR 1.86, 95% CI 1.07–3.24) relative to post-menopausal women who never used HRT but did not specify the formulation or hormone content [41]. Benson et al. [39] described an association between oestrogen but not progesterone-based HRT and meningioma incidence and a stronger association for use >5 years than <5 years. An association between meningioma diagnosis and HRT use was also described by Shu et al. (2019) [43] in an Asian population. In this case–control study, meningioma cases in women over 50 years of age were identified across three hospitals and matched with control patients who presented over the same time period to the study hospitals for a medical condition. Controls were selected using propensity matching in a 2:1 ratio. A total of 31.5% of cases and 27.9% of controls were ever-users of HRT, resulting in a higher risk of meningioma with HRT (OR 1.2; 95% CI 1.0–1.4). The strongest association was identified on a sensitivity analysis for long-term users of combination therapy (OR 1.5; 95% CI 1.2–4.4). Muskens et al. 2019 [42] identified an association between meningioma incidence and sole oestrogen HRT use per 5 years of use specifically in women with surgical menopause, RR 1.07 (1.01–1.15).

Two studies investigated the link between HRT use and PR/ER receptor status in meningioma and found no association [36,40]. There was no data on tumour molecular genetics and their impact on the relationship between meningiomas and HRT.

### 3.4. Broadly Defined Oral Contraceptives

Thirteen studies explored the association between meningioma and broadly defined hormonal contraceptives: 5 cohort studies and 8 case–control studies.

Seven studies found no positive association between oral contraceptives and meningiomas [36,39,41,45,47,48,49]. The majority of these studies did not specifically include information about the dose, formulation or duration of use of the oral contraceptive in their analysis, precluding detailed sub-analyses stratified according to those factors [36,39,41,48,49]. On the other hand, six studies did describe a positive association between oral contraceptive use and meningioma [27,40,42,44,46,50], but these studies were not necessarily methodologically stronger. For the studies which did identify associations between meningioma formation and oral contraceptive usage, the link was modest at best. Two studies reported an association between current use and meningioma development (rather than ever use) [44,46].

In terms of hormone receptor expression, one study found no association between PR status and oral contraceptive use [40], while another study described a strong association between oral contraceptive use and low PR expression within the tumour [36]. There was no data on tumour molecular genetics and their impact on the relationship between meningiomas and hormonal contraceptives.

## 4. Discussion

Despite being the most common central nervous system tumour, an understanding of the role of various factors on meningioma initiation and growth is still limited. As a result, it is a challenge for clinicians to understand how to apply the risks described in the literature regarding hormonal therapies to clinical decisions about individual patients. We sought to undertake this literature review to better describe the evidence concerning different forms of exogenous hormonal therapies and their impact on meningioma incidence and growth.

### 4.1. Potent Synthetic Progestins Demonstrate the Strongest and Most Consistent Evidence Linking Meningioma with Exogenous Hormones

We identified a number of studies that describe a clear risk of meningiomas with high-dose progestins. The highest risk described was with CPA [20,21,22,25], carrying an increased risk of meningioma of up to 19-fold compared with controls, followed by NOMAC [18,25] and CMA [25]. Risk was dose-dependent, and reversibility after withdrawal of the progestin was demonstrated. CPA increased the risk of multiple meningiomas [22,23] and anterior/middle skull base location of tumours [18,21,23,25]. However, in the papers we selected for this study, there was no strong link described between tumour grade and the use of high-dose progestins [22,23].

### 4.2. Emerging Evidence on the Role of Depot Medroxyprogesterone and Other Contraceptive Progestins

There is growing evidence that dMPA and other progestins may have an association with meningioma formation [27,28,29]. Much of this evidence is detailed in newer studies which have accurate drug dosage information. Longer duration of desogestrel also appears to carry an increased risk of meningioma [34]. In terms of clinical risk, the numbers needed to harm are 1152 for dMPA and 3020 for oral MPA, indicating that the absolute risks of these compounds for an individual patient are relatively low and that consideration of alternatives is of particular importance in patients with pre-existing risk factors for meningioma such as previous meningioma, history of radiation and diagnosis of neurofibromatosis type 2-related schwannomatosis. Roland et al. calculated that 67,287 women would need to be treated with desogestrel 75 ug to cause one meningioma requiring surgical treatment, compared to 518 women for CPA [34]. Again, this translates into a low risk for a patient with no prior risk factors for meningioma.

### 4.3. Evidence for Menopausal Hormonal Replacement and Broadly Defined Oral Contraceptive Use and Meningioma

Menopausal hormonal replacement and hormonal contraceptives involve a range of different exogenous hormone types and delivery routes. These include synthetic progestins and oestrogens. Compared with the high-dose progestin literature, the evidence base around menopausal hormonal replacement and broadly defined oral contraceptives is weak and not consistent enough to draw reliable conclusions from. For both MHT and oral contraceptives, some studies included in this review reported no association or negative association with meningioma, while others did find a small increased risk of meningioma. This inconsistency may be due to the fact that many of the original studies did not capture adequate data on dose, duration of use, drug preparation [36,40,41,44,45,46], or effectively control for confounding factors. As a result, further work needs to be done to identify low-risk or “safe” contraceptive options which could be preferentially offered to women with existing risk factors for meningioma formation. For example, Xiao et al. [29] did not find an increased risk of meningioma with combined oral contraceptives (COCs), intrauterine devices (IUDs), progestin-only pills (POP), or subdermal implantable contraceptives (SDI) compared with controls, suggesting that these might be possible alternatives to consider for women who cannot be prescribed dMPA [29].

### 4.4. Strengths and Limitations of Existing Evidence

There are multiple confounding factors that are problematic in the study of the link between exogenous hormonal treatments and meningioma development. These include issues such as surveillance bias, age, pregnancy, and the presence of other risk factors for meningioma development such as radiation exposure and genetic predisposition. These areas of possible bias and confounding were not uniformly addressed across all studies, particularly the larger, more historic cohort studies which form a significant proportion of the evidence base for broadly defined oral contraceptives. This is an important area of weakness which should be taken into account in the design of future studies.

We also acknowledge the heterogeneity of the datasets compared. For example, some studies specifically select surgically treated meningiomas whereas others include radiologically diagnosed meningiomas, the clinical significance of which is, without doubt, different. Furthermore, without having had an MRI to confirm absence of meningioma, patients in control groups may have incidental meningiomas which were undetected and could act as a confounding factor. In many studies, existence of meningioma in the control population was not formally excluded, which is reflected in a lower Newcastle-Ottawa score allocated to the study.

### 4.5. Clinical Studies Investigating the Link Between Exogenous Hormones and Meningioma Do Not Currently Include Strong Mechanistic Arms: Mechanistic Work Can Elucidate Relevant Molecular Pathways, Complementing Clinical Studies

The molecular classification of meningioma is an area of active research interest. The 2021 WHO classification added molecular features to the previously histologically based classification of meningiomas, with TERT promoter mutation and CDKN2A/B homozygous deletion signifying grade 3 tumours [2]. Analysis of genome-wide methylation patterns has resulted in an alternative way of classifying meningiomas, which may be effective at predicting recurrence and appears to cluster tumours with common mutational subtypes [52].

Key mutations implicated in meningioma biology include the NF2 tumour suppressor gene, SMO, TRAF7, PIK3CA, KLF4 and SMARCE1 [53,54], and these at the very least should be explored in future studies. NF2 mutation is the most common mutation in meningiomas, occurring in 40–60% of cases [55]. The NF2 gene on chromosome 22 encodes the cytoskeletal protein Merlin. The loss of Merlin either through NF2 mutation and/or chromosome 22 loss can drive meningioma growth via multiple mechanisms, including deregulation of the hippo signalling pathway with constitutive activation of YAP/TAZ and expression of growth promoting genes, also via loss of inhibition of phosphoinosidide 3-kinase (PI3K)/AKT signalling which results in activation of mammalian target of rapamycin complex 1 and 2 (mTORC1 and 2) and subsequent activation of target genes [56]. NF2 mutation is associated with more aggressive meningiomas. The PI3K/AKT/mTOR pathway can exhibit mutations independently of NF2 mutations in meningioma [57,58]. PIK3Ca is the gene that encodes PI3K; therefore, mutations can result in hyperactivation of the PI3K pathway. PIK3Ca mutations have been identified in meningiomas which tend to arise in the skull base [58]. Furthermore, KLF4, SMARCE1, SMO and TRAF7 are other commonly mutated genes in non-NF2 meningiomas [58]. TRAF7 is an E3 ubiquitin ligase which has been shown to occur in up to 25% of meningiomas and frequently co-occurs with KLF4 mutations [55]. SMO mutations were identified in 5% of a series of 300 meningiomas analysed using genomic approaches. SMO activating mutations in meningioma lead to activation of Hedgehog signalling, an oncogenic pathway described in multiple tumour types including medulloblastoma. SMO mutant meningiomas may be more closely associated with a skull base (medial/anterior) location, while NF2-associated meningiomas are more likely to be hemispheric, cerebellar or spinal [55].

Although some studies in our review included information about anatomic location and progesterone receptor status, none of the clinical studies we reviewed included integrated genomic analysis of the tumours from hormone exposed and non-exposed groups. Neither did they include key molecular profiling information such as Ki67 index, histological grade, methylation class or mutational status. Few studies described tumour location or hormone receptor status. Comprehensively linking these molecular and anatomic features with the drug exposure information is likely to be an important next step in this research field in order to delineate the molecular basis for the link between exogenous sex hormones and meningioma formation and potentially facilitate more effective risk stratification in patient groups.

Early basic science studies and translational studies have begun to explore the role of specific mutations and their link with CPA or progesterone receptor (PR) expression. For example, Supartoto et al. measured expression of PR and NF2 mRNA in serum samples from patients with orbito-cranial meningiomas and control participants and found lower serum PR and NF2 expression in patients with longer progesterone exposure, hypothesising that these could be linked by a common pathway to meningioma formation [59].

PI3KCa is an emerging candidate to link progesterone receptor signalling and meningioma [53]. PIK3Ca mutations have been correlated with hormone receptor expression in breast cancer, and progresterone is known to activate PI3K/AKT signalling in normal biological processes [60]. Portet et al. (2019) [61] identified PIK3Ca or AKT1 mutations in 33.3% of a series of 30 meningiomas associated with CPA therapy. Peyre et al. demonstrated a reworking of the mutational landscape in progestin-associated meningioma tumours and found a prominence of PIK3Ca-mutant skull base meningiomas amongst this group of tumours [62].

In order to further explore the link between PIK3Ca mutations and the progestin axis, Cômes et al. [63] tested the effects of CPA treatment in wild-type mice, Pik3ca- or NF2- mutant background mice. However, they found that although expression of mutant Pik3ca in the meninges (mouse model) or cell culture of primary arachnoid and dura mater cells led to meningioma formation or significant increased cellular proliferation respectively, CPA treatment did not have a significant additional tumorigenic effect. They concluded that CPA (in murine models and cell culture) does not appear to have a tumour-initiating effect, hypothesising that CPA effects observed clinically may be more closely related to a modification of cellular architecture. This may link to the possible promotional effect of progestin treatment on the tumour microenvironment, particularly vascularity and haemodynamic properties [64] akin to the hypothesis that meningioma growth during pregnancy is primarily or partly a haemodynamic phenomenon. Little is known about the interaction between hormone receptor status and the tumour microenvironment in meningiomas, nor how CPA and other exogenous/endogenous hormones affect this axis; an area for future study.

Cystic meningiomas are a rare subtype of meningioma, occurring in 3.5% of meningiomas described in a recent series [65]. There is a tendency for cystic meningiomas to be a higher grade than non-cystic meningiomas [65,66]. The observational studies reviewed in this article did not specifically assess the association between exogenous sex hormones and cystic meningiomas; however, a histopathological series looking at characterisation of 30 CPA-associated meningiomas found that half of the cohort had microcystic components, which is higher than would be expected in a standard series [61]. Further evaluation of the link between sex hormones and the development of cystic meningiomas would be of interest in future work.

### 4.6. Clinical Implications

Key clinical implications include avoidance of CPA and other highly potent progestins in patients with diagnosed meningioma; consideration of alternatives to dMPA in patients diagnosed with meningioma; avoidance of long-term progestin treatment where reasonable alternatives exist; initiation of surveillance MRI for patients on CPA and other highly potent progestins; and introducing individualised counselling where possible when starting treatment with exogenous sex hormones to clearly establish clinical history and presence of risk factors for meningioma to enable optimal choice of drug and formulation (Table 2).

## 5. Conclusions and Future Directions

In conclusion, the involvement of sex hormones in meningioma pathophysiology is an important area for future research, both to better understand the biological basis of the interaction but also to create clear guidelines for use of exogenous hormones in patients with other risk factors for meningioma or with an existing diagnosis of meningioma. This is likely to include further focused studies in specific patient groups with clearly defined doses and durations of exogenous hormones and with accompanying radiological and tissue diagnostic studies which incorporate relevant histological and genetic data, including Ki67, tumour grade, presence of key driver mutations and methylation status. Translational research also has the potential to shed light on other aspects of meningioma biology, for example, exploring factors underlying the predilection for the anterior skull base location amongst high-dose progestin-associated meningiomas and better understanding the role of sex hormone expression in meningioma formation and proliferation.

## Figures and Tables

**Table 1:** Hormonal therapies and their indications.

Hormone Content	Further Detail	Indication	Dose Pattern
Desogestrel	Synthetic progestin derived from testosterone.	Used in contraceptives (including as the “mini-pill”). Can also be used as part of HRT.	
Medroxyprogesterone acetate (MPA) (Depo-Provera)	Synthetic progestin. More resistant to metabolism and therefore has improved pharmacokinetic properties.	Used to treat secondary amenorrhoea, endometrial hyperplasia, abnormal uterine bleeding. Also used for pregnancy prevention.	Formulation: variable. Includes depot injection (3 monthly) (dMPA). May be advantageous for women who may not adhere to daily oral contraceptives.
Levonorgestrel	Synthetic progestin. Similar to progesterone.	Can be used as emergency contraception (“morning after pill”) and hormone therapy. Can be used as a hormonal contraceptive released from an intrauterine device, e.g., mirena coil	
Combined oestrogens and progestins		HRT option if patient has not had hysterectomy.	
Oestrogen only (e.g., 17-beta-oestradiol)		Used for HRT. Must be combined with a progestin if the patient still has a uterus.	Can be used as tablets, patches, gel or spray for HRT. Associated with risk of thrombosis. Patches can be changed every few days. Tablets/gels taken daily.
Cyproterone acetate (CPA)	Synthetic progestin with strong anti-androgen properties. Can be combined with ethinyl-oestradiol for certain indications and can be given as high dose (50–100 mg) and low dose (2 mg).	Indications include severe acne, hirsutism, gender affirming therapy, palliative treatment for prostate cancer.	
Nomegestrol acetate (NOMAC)	Synthetic progestin with mild-to-moderate anti-androgen properties.	Used in HRT and oral contraceptives.	
Chlormadinone acetate (CMA)	Synthetic progestin with moderate anti-androgen properties.	Used in HRT and oral contraceptives.	

**Table 2:** Proposed clinical considerations emerging from the review regarding different classes of exogenous hormones in the clinical setting.

Type of Exogenous Hormonal Therapy	Estimated Risk of Meningioma	Clinical Recommendation	Evidence Profile
CPA	Significant	Avoid in known diagnosis of meningioma Screening by MRI in patients on long-term CPA treatment	Strong
dMPA	Low absolute risk but may be significant in patients with other meningioma risk factors	Individualised counselling and risk stratification Consider alternatives in patients with existing meningioma risk factors	Emerging
Desogestrel	Low absolute risk but detectable over long duration of use	Individualised counselling and risk stratification Consider whether long-term use is essential, particularly in higher risk groups	Emerging
MHT	Risk unclear at this time; likely to be low	No specific guidance possible at this point	Weak
Broadly defined oral contraceptives	Risk unclear at this time; likely to be low	No specific guidance possible at this point	Weak

## Data Availability

No new data were created or analysed in this study. Data sharing is not applicable to this article.

## Supplementary Materials

The following supporting information can be downloaded at: https://www.mdpi.com/article/10.3390/jcm15145560/s1, File S1: Meningiomas and exogenous hormones; File S2: Newcastle-Ottawa Scale assessment.

## References

[1] Goldbrunner R., Stavrinou P., Jenkinson M.D., Sahm F., Mawrin C., Weber D.C., Preusser M., Minniti G., Lund-Johansen M., Lefranc F. (2021). EANO guideline on the diagnosis and management of meningiomas. Neuro Oncol..

[2] Louis D.N., Perry A., Wesseling P., Brat D.J., Cree I.A., Figarella-Branger D., Hawkins C., Ng H.K., Pfister S.M., Reifenberger G. (2021). The 2021 WHO Classification of Tumors of the Central Nervous System: A summary. Neuro Oncol..

[3] Sahm F., Aldape K.D., Brastianos P.K., Brat D.J., Dahiya S., von Deimling A., Giannini C., Gilbert M.R., Louis D.N., Raleigh D.R. (2025). CIMPACT-NOW update 8: Clarifications on molecular risk parameters and recommendations for WHO grading of meningiomas. Neuro Oncol..

[4] Abi Jaoude S., Peyre M., Degos V., Goutagny S., Parfait B., Kalamarides M. (2020). Validation of a scoring system to evaluate the risk of rapid growth of intracranial meningiomas in neurofibromatosis type 2 patients. J. Neurosurg..

[5] Brenner A.V., Sugiyama H., Preston D.L., Sakata R., French B., Sadakane A., Cahoon E.K., Utada M., Mabuchi K., Ozasa K. (2020). Radiation risk of central nervous system tumors in the Life Span Study of atomic bomb survivors, 1958–2009. Eur. J. Epidemiol..

[6] Wiemels J., Wrensch M., Claus E.B. (2010). Epidemiology and etiology of meningioma. J. Neurooncol..

[7] Shao C., Bai L.P., Qi Z.Y., Hui G.Z., Wang Z. (2014). Overweight, obesity and meningioma risk: A meta-analysis. PLoS ONE.

[8] Takahashi H., Cornish A.J., Sud A., Law P.J., Disney-Hogg L., Calvocoressi L., Lu L., Hansen H.M., Smirnov I., Walsh K.M. (2019). Mendelian randomization provides support for obesity as a risk factor for meningioma. Sci. Rep..

[9] Kerschbaumer J., Freyschlag C.F., Stockhammer G., Taucher S., Maier H., Thomé C., Seiz-Rosenhagen M. (2016). Hormone-dependent shrinkage of a sphenoid wing meningioma after pregnancy: Case report. J. Neurosurg..

[10] Custer B.S., Koepsell T.D., Mueller B.A. (2002). The association between breast carcinoma and meningioma in women. Cancer.

[11] Lopez-Rivera V., Zhu P., Dono A., Lee S., Chen P., Ballester L., Sheth S., Esquenazi Y. (2020). Increased Risk of Subsequent Meningioma Among Women with Malignant Breast Cancer. World Neurosurg..

[12] Guresci S., Aydogdu O.B., Secen A.E., Uzel B. (2025). Assessing the predictive value of Ki-67 and progesterone receptor algorithms for recurrence and disease-free survival in meningiomas. Ann. Diagn. Pathol..

[13] Portet S., Banor T., Bousquet J., Simonneau A., Flores M., Ingrand P., Milin S., Karayan-Tapon L., Bataille B. (2020). New Insights into Expression of Hormonal Receptors by Meningiomas. World Neurosurg..

[14] Kuroi Y., Matsumoto K., Shibuya M., Kasuya H. (2018). Progesterone Receptor Is Responsible for Benign Biology of Skull Base Meningioma. World Neurosurg..

[15] Cyproterone Acetate: New Advice to Minimise Risk of Meningioma. https://www.gov.uk/drug-safety-update/cyproterone-acetate-new-advice-to-minimise-risk-of-meningioma.

[16] Cyproterone-Containing Medicinal Products—Referral. https://www.ema.europa.eu/en/medicines/human/referrals/cyproterone-containing-medicinal-products.

[17] Page M.J., McKenzie J.E., Bossuyt P.M., Boutron I., Hoffmann T.C., Mulrow C.D., Shamseer L., Tetzlaff J.M., Akl E.A., Brennan S.E. (2021). The PRISMA 2020 statement: An updated guideline for reporting systematic reviews. BMJ.

[18] Nguyen P., Roland N., Neumann A., Hoisnard L., Passeri T., Duranteau L., Coste J., Froelich S., Zureik M., Weill A. (2024). Prolonged use of nomegestrol acetate and risk of intracranial meningioma: A population-based cohort study. Lancet Reg. Health Eur..

[19] Roland N., Nguyen P., Neumann A., Hoisnard L., Passeri T., Duranteau L., Coste J., Froelich S., Zureik M., Weill A. (2025). Prolonged use of chlormadinone acetate and risk of intracranial meningioma: A population-based cohort study. Eur. J. Neurol..

[20] Mikkelsen A.P., Greiber I.K., Scheller N.M., Hilden M., Lidegaard Ø. (2022). Cyproterone acetate and risk of meningioma: A nationwide cohort study. J. Neurol. Neurosurg. Psychiatry.

[21] Weill A., Nguyen P., Labidi M., Cadier B., Passeri T., Duranteau L., Bernat A.L., Yoldjian I., Fontanel S., Froelich S. (2021). Use of high dose cyproterone acetate and risk of intracranial meningioma in women: Cohort study. BMJ.

[22] Samarut E., Lugat A., Amelot A., Scharbarg E., Hadjadj S., Primot C., Loussouarn D., Thillays F., Buffenoir K., Cariou B. (2021). Meningiomas and cyproterone acetate: A retrospective, monocentric cohort of 388 patients treated by surgery or radiotherapy for intracranial meningioma. J. Neurooncol..

[23] Champeaux-Depond C., Weller J., Froelich S., Sartor A. (2021). Cyproterone acetate and meningioma: A nationwide-wide population based study. J. Neurooncol..

[24] Gil M., Oliva B., Timoner J., Maciá M.A., Bryant V., de Abajo F.J. (2011). Risk of meningioma among users of high doses of cyproterone acetate as compared with the general population: Evidence from a population-based cohort study. Br. J. Clin. Pharmacol..

[25] Hoisnard L., Laanani M., Passeri T., Duranteau L., Coste J., Zureik M., Froelich S., Weill A. (2022). Risk of intracranial meningioma with three potent progestogens: A population-based case-control study. Eur. J. Neurol..

[26] Nota N.M., Wiepjes C.M., de Blok C.J.M., Gooren L.J.G., Peerdeman S.M., Kreukels B.P.C., den Heijer M. (2018). The occurrence of benign brain tumours in transgender individuals during cross-sex hormone treatment. Brain.

[27] Chen S., Jugl S., Jackson L., Rahman M., Antonelli P.J., Bruggeman B., Winterstein A.G. (2026). Risk of intracranial meningioma requiring surgical intervention among recent hormonal contraceptives users. J. Neurooncol..

[28] Griffin R., Arend R. (2025). A Matched Case-Control Study Examining the Association Between Exposure to Depot Medroxyprogesterone Acetate and Cerebral Meningioma Using an Active Comparator. Curr. Oncol..

[29] Xiao T., Kumar P., Lobbous M., Yogi-Morren D., Soni P., Recinos P.F., Kshettry V.R. (2025). Depot Medroxyprogesterone Acetate and Risk of Meningioma in the US. JAMA Neurol..

[30] Tettamanti G., Shu X., Mogensen H., Kopp Kallner H., Mathiesen T., Feychting M. (2026). Hormonal contraceptives and the risk of meningioma: A Swedish register-based case-control study. Neuro Oncol..

[31] Reynolds L.M., Arend R., Griffin R.L. (2025). The Association Between Medroxyprogesterone Acetate Exposure and Cerebral Meningioma Among a Medicaid Population. Epidemiologia.

[32] Frey C., Sodhi M., Fatehi M., Kezouh A., Etminan M. (2025). Use of medroxyprogesterone acetate and risk of meningiomas: A comparative safety study. Expert Opin. Drug Saf..

[33] Roland N., Neumann A., Hoisnard L., Duranteau L., Froelich S., Zureik M., Weill A. (2024). Use of progestogens and the risk of intracranial meningioma: National case-control study. BMJ.

[34] Roland N., Kolla E., Baricault B., Dayani P., Duranteau L., Froelich S., Zureik M., Weill A. (2025). Oral contraceptives with progestogens desogestrel or levonorgestrel and risk of intracranial meningioma: National case-control study. BMJ.

[35] Pourhadi N., Meaidi A., Friis S., Torp-Pedersen C., Mørch L.S. (2023). Menopausal hormone therapy and central nervous system tumors: Danish nested case-control study. PLoS Med..

[36] Custer B., Longstreth W.T., Phillips L.E., Koepsell T.D., Van Belle G. (2006). Hormonal exposures and the risk of intracranial meningioma in women: A population-based case-control study. BMC Cancer.

[37] Dresser L., Yuen C.A., Wilmington A., Walker M., Vogel T.J., Merrell R.T., Kamson D.O. (2020). Estrogen hormone replacement therapy in incidental intracranial meningioma: A growth-rate analysis. Sci. Rep..

[38] Andersen L., Friis S., Hallas J., Ravn P., Schrøder H.D., Gaist D. (2013). Hormone replacement therapy increases the risk of cranial meningioma. Eur. J. Cancer.

[39] Benson V.S., Pirie K., Green J., Casabonne D., Beral V. (2008). Million Women Study Collaborators. Lifestyle factors and primary glioma and meningioma tumours in the Million Women Study cohort. Br. J. Cancer.

[40] Korhonen K., Raitanen J., Isola J., Haapasalo H., Salminen T., Auvinen A. (2010). Exogenous sex hormone use and risk of meningioma: A population-based case-control study in Finland. Cancer Causes Control.

[41] Jhawar B.S., Fuchs C.S., Colditz G.A., Stampfer M.J. (2003). Sex steroid hormone exposures and risk for meningioma. J. Neurosurg..

[42] Muskens I.S., Wu A.H., Porcel J., Cheng I., Le Marchand L., Wiemels J.L., Setiawan V.W. (2019). Body mass index, comorbidities, and hormonal factors in relation to meningioma in an ethnically diverse population: The Multiethnic Cohort. Neuro Oncol..

[43] Shu X., Jiang Y., Wen T., Lu S., Yao L., Meng F. (2019). Association of hormone replacement therapy with increased risk of meningioma in women: A hospital-based multicenter study with propensity score matching. Asia Pac. J. Clin. Oncol..

[44] Claus E.B., Calvocoressi L., Bondy M.L., Wrensch M., Wiemels J.L., Schildkraut J.M. (2013). Exogenous hormone use, reproductive factors, and risk of intracranial meningioma in females. J. Neurosurg..

[45] Wigertz A., Lönn S., Mathiesen T., Ahlbom A., Hall P., Feychting M. (2006). Swedish Interphone Study Group. Risk of brain tumors associated with exposure to exogenous female sex hormones. Am. J. Epidemiol..

[46] Michaud D.S., Gallo V., Schlehofer B., Tjønneland A., Olsen A., Overvad K., Dahm C.C., Kaaks R., Lukanova A., Boeing H. (2010). Reproductive factors and exogenous hormone use in relation to risk of glioma and meningioma in a large European cohort study. Cancer Epidemiol. Biomark. Prev..

[47] Edwards E., Tsai K., Pappu S., Gaddey H., Nofzinger T.B., Vachon E., McConomy B. (2026). Incidence of meningioma in women with a history of combined oral contraceptive pill use and polycystic ovary syndrome. Womens Health.

[48] Anic G.M., Madden M.H., Nabors L.B., Olson J.J., LaRocca R.V., Thompson Z.J., Pamnani S.J., Forsyth P.A., Thompson R.C., Egan K.M. (2014). Reproductive factors and risk of primary brain tumors in women. J. Neurooncol..

[49] Hatch E.E., Linet M.S., Zhang J., Fine H.A., Shapiro W.R., Selker R.G., Black P.M., Inskip P.D. (2005). Reproductive and hormonal factors and risk of brain tumors in adult females. Int. J. Cancer.

[50] Harland T.A., Freeman J.L., Davern M., McCracken D.J., Celano E.C., Lillehei K., Olson J.J., Ormond D.R. (2018). Progesterone-only contraception is associated with a shorter progression-free survival in premenopausal women with WHO Grade I meningioma. J. Neurooncol..

[51] Devalckeneer A., Aboukais R., Faisant M., Bourgeois P., Quentin V.M., Maurage C.A., Escande F., Lejeune J.P. (2022). Progestin-related WHO grade II meningiomas behavior-a single-institution comparative case series. Neurosurg. Rev..

[52] Sahm F., Schrimpf D., Stichel D., Jones D.T.W., Hielscher T., Schefzyk S., Okonechnikov K., Koelsche C., Reuss D.E., Capper D. (2017). DNA methylation-based classification and grading system for meningioma: A multicentre, retrospective analysis. Lancet Oncol..

[53] Abikenari M., Regev A., Himic V., Choi J., Jeyaretna S., Fountain D.M., Lim M. (2025). The hormonal nexus in PIK3CA-mutated meningiomas: Implications for targeted therapy and clinical trial design. J. Neurooncol..

[54] Alkhatib M., Hua L., Beyer F., Prilop I., Podlesek D., Jeyaretna S., Fujio S., Zolal A., Günther L., Cicek B. (2025). Molecular landscape and clinical correlates of olfactory groove meningiomas: A multi-institutional study. J. Neurosurg..

[55] Clark V.E., Erson-Omay E.Z., Serin A., Yin J., Cotney J., Ozduman K., Avşar T., Li J., Murray P.B., Henegariu O. (2013). Genomic analysis of non-NF2 meningiomas reveals mutations in TRAF7, KLF4, AKT1, and SMO. Science.

[56] Lee S., Karas P.J., Hadley C.C., Bayley V.J.C., Khan A.B., Jalali A., Sweeney A.D., Klisch T.J., Patel A.J. (2019). The Role of Merlin/NF2 Loss in Meningioma Biology. Cancers.

[57] Brastianos P.K., Horowitz P.M., Santagata S., Jones R.T., McKenna A., Getz G., Ligon K.L., Palescandolo E., Van Hummelen P., Ducar M.D. (2013). Genomic sequencing of meningiomas identifies oncogenic SMO and AKT1 mutations. Nat. Genet..

[58] Abedalthagafi M., Bi W.L., Aizer A.A., Merrill P.H., Brewster R., Agarwalla P.K., Listewnik M.L., Dias-Santagata D., Thorner A.R., Van Hummelen P. (2016). Oncogenic PI3K mutations are as common as AKT1 and SMO mutations in meningioma. Neuro Oncol..

[59] Supartoto A., Sasongko M.B., Respatika D., Mahayana I.T., Pawiroranu S., Kusnanto H., Sakti D.H., Nurlaila P.S., Heriyanto D.S., Haryana S.M. (2019). Relationships Between Neurofibromatosis-2, Progesterone Receptor Expression, the Use of Exogenous Progesterone, and Risk of Orbitocranial Meningioma in Females. Front. Oncol..

[60] Liu H., Yu L., Ding Y., Peng M., Deng Y. (2023). Progesterone Enhances the Invasion of Trophoblast Cells by Activating PI3K/AKT Signaling Pathway to Prevent Preeclampsia. Cell Transplant..

[61] Portet S., Naoufal R., Tachon G., Simonneau A., Chalant A., Naar A., Milin S., Bataille B., Karayan-Tapon L. (2019). Histomolecular characterization of intracranial meningiomas developed in patients exposed to high-dose cyproterone acetate: An antiandrogen treatment. Neurooncol. Adv..

[62] Peyre M., Gaillard S., De Marcellus C., Giry M., Bielle F., Villa C., Boch A.L., Loiseau H., Baussart B., Cazabat L. (2018). Progestin- associated shift of meningioma mutational landscape. Ann. Oncol. J. Eur. Soc. Med. Oncol..

[63] Cômes P.C., Le Van T., Tran S., Huard S., Abi-Jaoude S., Venot Q., Marijon P., Boetto J., Blouin A., Bielle F. (2023). Respective roles of Pik3ca mutations and cyproterone acetate impregnation in mouse meningioma tumorigenesis. Cancer Gene Ther..

[64] Kalamarides M., Peyre M. (2017). Dramatic Shrinkage with Reduced Vascularization of Large Meningiomas After Cessation of Progestin Treatment. World Neurosurg..

[65] Boukobza M., Cebula H., Pop R., Kouakou F., Sadoun A., Coca H.A., Polivka M., Diemidio P., Ganau M., George B. (2016). Cystic meningioma: Radiological, histological, and surgical particularities in 43 patients. Acta Neurochir..

[66] Go K.O., Lee K., Heo W., Lee Y.S., Park Y.S., Kim S.K., Lee J.H., Jung J.M. (2018). Cystic Meningiomas: Correlation between Radiologic and Histopathologic Features. Brain Tumor Res. Treat..

